# Exploring microtubule dynamics in Alzheimer's disease: Longitudinal assessment using [^11^C]MPC‐6827 PET imaging in rodent models of Alzheimer's‐related pathology

**DOI:** 10.1002/alz.14083

**Published:** 2024-07-05

**Authors:** Naresh Damuka, Riley E. Irmen, Ivan Krizan, Mack Miller, Krishna K. Gollapelli, Bhuvanachandra Bhoopal, Ojasvi Deep, Avinash Bansode, Samuel N. Lockhart, Miranda E. Orr, Pooja Jadiya, Nagaraju Bashetti, J. V. Shanmukha Kumar, Akiva Mintz, Christopher T. Whitlow, Suzanne Craft, Shannon L. Macauley, Kiran K. Solingapuram Sai

**Affiliations:** ^1^ Department of Radiology Wake Forest School of Medicine Winston‐Salem North Carolina USA; ^2^ Department of Physiology University of Kentucky Lexington Kentucky USA; ^3^ Department of Gerontology and Geriatric Medicine Wake Forest School of Medicine Winston‐Salem North Carolina USA; ^4^ Department of Chemistry Koneru Lakshmaiah Education Foundation Vijayawada Andhra Pradesh India; ^5^ Department of Radiology Columbia University Medical Center New York New York USA

**Keywords:** microtubules, PET imaging, PET radioligands, radiochemistry, rodent models

## Abstract

**INTRODUCTION:**

Microtubule (MT) stability is crucial for proper neuronal function. Understanding MT dysregulation is critical for connecting amyloid beta (Aβ) and tau‐based degenerative events and early changes in presymptomatic Alzheimer's disease (AD). Herein we present positron emission tomography (PET) imaging properties of our MT‐PET radiotracer, [^11^C]MPC‐6827, in multiple established AD mouse models.

**METHODS:**

Longitudinal PET, biodistribution, autoradiography, immunohistochemistry, and behavioral studies were conducted at multiple time points in APPswe/PSEN1dE9 (APP/PS1), P301S‐PS19 (P301S), 5xFAD, and age‐matched control mice.

**RESULTS:**

Longitudinal [^11^C]MPC‐6827 brain imaging showed significant increases in APP/PS1, P301S, and 5xFAD mice compared to controls. Longitudinal MT‐PET correlated positively with biodistribution, autoradiography, and immunohistochemistry results and negatively with behavior data.

**DISCUSSION:**

Our study demonstrated significant longitudinal [^11^C]MPC‐6827 PET increases in multiple AD mouse models for the first time. Strong correlations between PET and biomarker data underscored the interplay of MT destabilization, amyloid, and tau pathology in AD. These results suggest [^11^C]MPC‐6827 PET as a promising tool for monitoring MT dysregulation early in AD progression.

**Highlights:**

- Longitudinal positron emission tomography (PET) imaging studies using [^11^C]MPC‐6827 in multiple established Alzheimer's disease (AD) mouse models revealed an early onset of microtubule dysregulation, with significant changes in brain radiotracer uptake evident from 2 to 4 months of age.
- Intra‐group analysis showed a progressive increase in microtubule dysregulation with increasing AD burden, supported by significant correlations between PET imaging data and biodistribution, autoradiography, and molecular pathological markers.
- [^11^C]MPC‐6827 PET imaging demonstrated its efficacy in detecting early microtubule alterations preceding observable behavioral changes in AD mouse models, suggesting its potential for early AD imaging.
- The inclusion of the 5xFAD mouse model further elucidated the impact of amyloid beta (Aβ) toxicity on inducing tau hyperphosphorylation‐mediated microtubule dysregulation, highlighting the versatility of [^11^C]MPC‐6827 in delineating various aspects of AD pathology.
- Our study provides immediate clarity on high uptake of the microtubule‐based radiotracer in AD brains in a longitudinal setting, which directly informs clinical utility in Aβ/tau‐based studies.

## 1 BACKGROUND

Alzheimer's disease (AD) is an incurable neurodegenerative disorder and its hallmarks including progressive cognitive decline, memory impairment, and behavioral changes, devastate individual patients, their families, and health care systems.^1^ Over 13 million patients worldwide AD; with extended longevity, this number is projected to exceed 88 million by 2050.^2^, ^3^ The need to define AD pathophysiology and develop effective early detection strategies and therapeutic interventions is urgent.

Current research focuses on amyloid beta (Aβ) aggregation into amyloid plaques,^4^ intraneuronal inclusions in the form of neurofilaments, hyperphosphorylated tau protein (p‐tau),^5^ synaptic dysfunction,^6^ neuroinflammation,^7^ structural changes in cerebrovascular function, impairments in cerebral glucose uptake, and cerebral blood flow.^8^ Remarkably, these pathological processes begin decades before the onset of clinical symptoms, opening a window for early diagnosis and creating a need for early intervention strategies. There is a critical need to develop early biomarkers potential to delineate different stages of disease across the AD continuum.^9^ The AT(N) framework, introduced by the National Institute on Aging (NIA) and the Alzheimer's Association (AA), is recognized as a valuable system for classifying AD biomarkers^10^: A = Aβ, T = tau, and N = neurodegeneration.^11^ These markers can be assessed through neuroimaging techniques including magnetic resonance imaging (MRI) and positron emission tomography (PET), as well as analysis of plasma or cerebrospinal fluid (CSF) biomarkers.^5^, ^12^, ^13^, ^14^ Although these imaging and fluid biomarkers have provided significant insights into AD pathology relative to Aβ and tau pathology, additional biomarkers are needed to understand how Aβ and tau alter neuronal integrity, structure, and function for early diagnosis and staging of AD.^15^

Microtubules (MTs) play a critical role in axonal transport, neuron structure, and plasticity, and when altered, lead to neurodegeneration. MT abnormalities are heavily implicated in AD pathology.^16^ Oligomeric Aβ, a component of amyloid plaques, disrupts synaptic function and dendritic morphology, which contributes to learning and memory. MTs are integral to these processes, with dynamic MTs regulating dendritic spine morphology and synaptic plasticity, whereas stable MTs contribute to dendritic arbor complexity.^17^ The MT‐associated protein tau stabilizer of MTs is important for neuronal function, and plays a crucial role in MT dynamics. Although tau hyperphosphorylation is linked to MT destabilization, its role in regulating MT dynamics appears complex. Tau influence on MT dynamics in axons and dendrites differs, impacting neuronal structure and plasticity.^18^ Furthermore, tau‐independent mechanisms also affect MT dynamics, particularly in dendritic spines, where MT invasion and calcium signaling play roles in synaptic plasticity. Aβ‐induced transmembrane signaling may directly influence MTs, affecting their stability and function.^19^ Understanding of the intricate interplay between tau, Aβ, and MTs offers potential insights into AD pathology and aids in therapeutic interventions targeting MT‐related changes.

PET is a powerful noninvasive imaging modality known for its high sensitivity and quantitative analysis.^20^ PET ligands that target MTs offer promising avenues early pathological signs of AD.^21^ Because these changes emerge years before clinical symptoms, PET offer a platform to monitor real‐time functional changes to diagnose and monitor AD progression longitudinally.

In the context of MT‐PET, our group is the first to design, develop, and evaluate the blood–brain barrier (BBB)–penetrating MT‐radioligand [^11^C]MPC‐6827.^22^ Initial evaluations in rodent models have shown its high affinity for destabilized MTs, and its excellent pharmacokinetics in both rodent and nonhuman primate (NHP) brains.^21^, ^23^, ^24^ We assessed [^11^C]MPC‐6827 uptake in NHPs exhibiting AD‐like symptoms, and the results support its potential for clinical translation.^25^ Because PET provides functional insights into AD pathogenesis, we use [^11^C]MPC‐6827 PET to study MT changes in established rodent models of AD‐related pathology in a longitudinal setting. We hypothesize that [^11^C]MPC‐6827 PET would quantify MT‐based changes before the onset of amyloid plaques and neurofibrillary tangles or changes in behavioral effects. In this current study, [^11^C]MPC‐6827 PET was characterized longitudinally in a Aβ‐overexpressing APP/PS1 and tau‐overexpressing P301S transgenic mouse models to track MT changes over time in vivo, ex vivo, and in vitro, and correlating them with changes in Aβ, tau, and behavior. We also included 5xFAD mouse model to enhance the scientific rigor of our study, illuminating how Aβ and tau pathology affect MT dynamics using [^11^C]MPC‐6827 PET.

## 2 METHODS

### 2.1 Animals

For longitudinal studies, we selected APPswe/PSEN1dE9 (APP/PS1; ID:034832‐JAX) and P301S PS19 (P301S; ID:008169‐JAX) mice (both male and female) to investigate the temporal progression of Aβ and tau pathology. The APP/PS1 mouse model, expressing chimeric amyloid precursor protein and mutant presenilin 1, allowed us to examine Aβ‐related changes. In this model, Aβ deposition starts at 6 months in the hippocampus and cortex and progresses with age. Behavioral deficits occur as early as 9–12 months in this model.^26^ The transgenic P301S PS19 model of tauopathy provides insights into tau pathology, with early tau pathology detected at ≈3–4 months. Age‐related cognitive decline and motor deficits progress over time, with ≈80% mortality by 12 months.^27^ These models collectively serve as crucial tools for elucidating the MT dysregulation relative to Aβ and tau pathology. In addition, to validate the interconnection of MT dynamics with intraneuronal Aβ_42_‐induced neurodegeneration^28^ and tau hyper‐phosphorylation,^29^ we performed PET imaging in the 5xFAD mouse model (ID:034840‐JAX). All the transgenic and age‐matched wild‐type (WT) littermates were obtained from Jackson Laboratory (Bar Harbor, ME, USA) at 4 weeks of age and were assigned for tubulin assay, PET/CT imaging, autoradiography, behavioral, or biodistribution studies. They were maintained under standard conditions with ad libitum access to food and water. All animal procedures were conducted in compliance with the Institutional Animal Care and Use Committee (IACUC)–approved protocols and adhered to the guidelines for the care and utilization of research animals established by Wake Forest School of Medicine Animal Studies.

**RESEARCH IN CONTEXT**

1. **Systematic review**: A comprehensive evaluation of Alzheimer's disease (AD) pathology highlights the intricate relationship between amyloid beta aggregation, tau protein hyperphosphorylation, and microtubule dysregulation, offering a roadmap for future diagnostic and therapeutic endeavors.
2. **Interpretation**: Analysis of longitudinal positron emission tomography (PET) imaging studies utilizing [^11^C]MPC‐6827 underscores early microtubule dysregulation in AD mouse models, indicating the potential of this novel radioligand for early detection and dynamic monitoring of AD progression.
3. **Future directions**: Validation of [^11^C]MPC‐6827 PET imaging in human clinical trials holds promise for detecting MT alterations in patients with AD.

### 2.2 Radiotracer synthesis

[^11^C]MPC‐6827 radiochemistry was carried out following our previously reported methods^22^ at the Wake Forest Translational Imaging Program PET Center. Briefly, desmethyl MPC‐6827 (ABX, Radeberg, Germany) was radiolabeled at the phenolic site with [^11^C]MeI (generated from [^11^C]CO_2_) at 80°C for 5 min in dimethylformamide (DMF) to yield [^11^C]MPC‐6827. The final radiotracer was formulated in a physiologic saline/ethanol solution (90:10, v:v) for all further rodent studies.

### 2.3 PET/CT imaging

PET/CT imaging was conducted on APP/PS1, P301S, and 5xFAD mice (*n* = 7 per group) using the TriFoil microPET/CT scanner.^30^ Anesthetized mice underwent dynamic 0–60 min brain PET scans after tail vein injection of [^11^C]MPC‐6827 (18.1 ± 0.01 MBq). APP/PS1 mice were imaged at 2, 3, 5, 7, 10, 13, and 19 months of age, whereas P301S mice were imaged at 2, 3, 6, 7, and 12 months. 5xFAD mice were imaged at 12 months of age. Image analysis was performed using PMOD software (PMOD technologies, v‐4.3 Switzerland), with regions of interest (ROIs) defined to plot standard uptake values (SUVs) and time activity curves (TACs) for whole brain.

### 2.4 Biodistribution studies

A subset of mice from the APP/PS1 and P301S models (*n* = 8 for TG and WT groups) were used for ex vivo biodistribution studies at the same PET imaging time‐points. Mice were euthanized 60 min post‐injection of the radiotracer as per the protocol established in our previous studies.^22^, ^31^ After [^11^C]MPC‐6827 was administered, all standard organs including blood, heart, liver, lung, spleen, pancreas, kidneys, and muscle were excised and weighed, and the associated radioactivity was measured and decay‐corrected using γ (gamma) counter, following our previously reported protocols.^30^, ^32^, ^33^ The percentage dose per gram (%ID/g) was calculated for each tissue after decay correction.

### 2.5 Autoradiography

Another cohort of APP/PS1, P301S, and 5xFAD mice (*n* = 3 per TG and WT groups) underwent ex vivo autoradiography (ARG). Brain sections from APP/PS1 mice at 2, 6, 12, and 18 months of age; P301S mice at 2, 6, and 12 months of age; and 5xFAD mice at 12 months of age were exposed to [^11^C]MPC‐6827 (0.5 ± 1 MBq/section). Autoradiography images were captured and analyzed using a GE Amersham Typhoon scanner (25 μm pixel size) and ImageQuant TL 8.2 and MCID core 7.1 software. Results from manually drawn ROIs were reported as photo‐stimulated luminescence signals per square millimeter (PSL/mm^2^).

### 2.6 Behavioral study: Nest building

Nest‐building behaviors were conducted with APP/PS1, P301S (*n* = 6/group) at 2, 6, and 12 months of age and 5xFAD mice (*n* = 3/group) at 12 months of age. Mice were individually transferred to clean cages 1 day prior to the nesting experiment. Any nesting materials present in the cage were completely removed, and ≈20 g of cotton nestlets (5 cm × 5 cm pressed cotton squares) were introduced into each cage. Mice were given 24 h to construct their nests. The following day, any unshredded cotton pads were weighed, and the initial weight was subtracted to determine each mouse's netlet construction as a measure of their cognitive activity.

### 2.7 Immunohistochemistry

#### 2.7.1 Quantification for Aβ deposition

Male and female APP/PS1 and WT mice from 2 to 18 months of age were anesthetized with isoflurane and transcardially perfused with cold phosphate‐buffered saline (PBS) with 0.3% heparin. The brains were removed and fixed in 4% paraformaldehyde (PFA) for at least 48 h at 4°C. After being fixed in 4% PFA, the brains were cryoprotected in 30% sucrose at 4°C until ready to be sectioned. Sectioning was performed using a freezing microtome at 40 μm, and sections were stored in cryoprotectant at −10°C. Three serial sections (300 μm apart) through the anterior and posterior hippocampus were selected for immunohistochemistry for Aβ deposition. Staining and quantification was completed as described previously.^34^ Free‐floating sections were immunostained for Aβ deposition using a biotinylated, HJ3.4 antibody (anti‐Aβ1‐13, mouse monoclonal antibody, a generous gift from the Holtzman Lab, Washington University, St. Louis, MO). Aβ deposition staining was developed with a Vectastain ABC kit (PK‐6100, Vector Labs) and DAB reaction (ICN 980681, Fisher Scientific). The brain sections were imaged using the Wake Forest Imaging Core NanoZoomer slide scanner. The images were quantified using ImageJ, where they were converted to 8‐bit grayscale and thresholded to highlight HJ3.4 specific staining. Percent occupied by HJ3.4 stain was quantified by a blinded researcher throughout the hippocampus and cortex. Statistical significance was determined at each time point using Student's *t*‐test and represented as means ± standard error of the mean (SEM).

#### 2.7.2 Quantification for phosphorylated tau

Male and female P301S PS19 and WT mice from 2 to 13 months of age were anesthetized with isoflurane and transcardially perfused with heparinized PBS. The mouse brains were then dissected from the skull and fixed in 4% PFA for 48 h at 4°C. After being fixed in PFA, the brains were cryoprotected in 30% sucrose at 4°C until ready to be sectioned. Sectioning was performed using a freezing microtome at 40 μm, and sections were stored in cryoprotectant at −10°C. Serial sections (300 μm apart) through the anterior‐posterior aspect of the hippocampus were immunostained for hyperphosphorylated tau deposition using a biotinylated, AT8 monoclonal antibody (anti‐tau pSer202, Thr205, Thermofisher). The AT8 antibody stains specifically for paired helical filaments tau and recognizes phosphorylation at serine 202 and threonine 205. The stain was developed using a Vectastain ABC kit (PK‐6100, Vector Labs) and DAB reaction (ICN 980681, Fisher Scientific). The brain sections were imaged using a NanoZoomer slide scanner (Hamamatsu Photonics, Wake Forest Imaging Core) and converted to 8‐bit grayscale and thresholded to highlight AT8 specific staining. Percent occupied by AT8 was quantified by a blinded researcher throughout the hippocampus and cortex. Statistical significance was determined at each time point using Student's *t*‐test and represented as means ± SEM (standard error of the mean).

### 2.8 Cytoskeleton‐based tubulin assay

To determine the ratio of free to bound tubulin in different age groups of APP/PS1 and P301S mice (*n* = 3/group), we employed a commercially available MT‐based assay kit (Cytoskeleton, Inc. BK038, Denver, CO, USA) as described previously.^24^, ^35^ In brief, 100 mg of brain sections obtained from euthanized mice were immediately immersed in a MT stabilization buffer at 37°C. Tissue lysates were prepared using a homogenizer maintained at 37°C. These lysates were then subjected to a centrifugation step at 1000 × *g* for 5 min, resulting in the separation of bound tubulin in the low‐speed pellet fraction. The supernatant samples were further re‐centrifuged at a high speed of 100,000 × *g* for 1 h at 37°C to segregate free tubulins.

After bound and free tubulin fragments were isolated, these samples were loaded onto a 12% sodium dodecyl sulfate‐polyacrylamide gel electrophoresis (SDS‐PAGE) according to the manufacturer's recommendations. Subsequently, the separated proteins were transferred onto a nitrocellulose membrane, followed by blocking the membrane using 5% non‐fat milk. The membranes were then incubated with a primary α/β tubulin antibody (1:2000) at 4°C overnight. Afterward, the membranes were subjected to washing steps with Tris buffered saline‐tween 20 (TBST) (3 times for 15 min each) at room temperature (RT) with agitation. Following the washes, the membranes were exposed to an HRP‐conjugated anti‐sheep secondary antibody (1:10,000) for an additional hour and subjected to further washing in TBST (3 times for 15 min each) at RT. Finally, the tubulin bands were visualized using an enhanced Chemiluminescence kit, and images were captured using an Amersham Imager (600—IA600). Tubulin concentrations were measured using Molecular ImageJ analysis software.

### 2.9 Statistical analysis

GraphPad Prism program (version 9; GraphPad Software La Jolla, CA, USA) was used for all statistical evaluations. The image analysis team was blinded to all rodent information, including type of mice, age, and sex. All the established standard operating procedures and protocols were followed for [^11^C]MPC6827 radiochemistry, quality control (QC), quality assurance (QA), scanning, and animal experiments. For in vivo PET images, we compared whole‐brain radiotracer uptake between the wild and transgenic groups using two‐way, repeated‐measures analysis of variance (ANOVA). Correlations between the two parameters were tested by Pearson (*r*). Šídák's multiple comparisons test was performed to compare the transgenic group at each time point with the control group. *P*‐values of *p < 0.01, **p < 0.001, ****p < 0.0001, and ****p < 0.0001 were interpreted as statistically significant.

## 3 RESULTS

### 3.1 [^11^C]MPC‐6827 radiochemistry

The total radiochemistry time from end‐of‐bombardment (EOB) from cyclotron to final radiotracer elution to the sterile final product vial was ≈50 min, including [^11^C]MeI generation, radiolabeling, semi‐prep HPLC separation, C18 SepPak purification, and formulation steps. [^11^C]MPC‐6827 showed a high molar activity of 100 ± 25 GBq/μmol, chemical and radiochemical purity was >99%, and a cold mass of 3–5 μg/batch, decay corrected to end of synthesis (EOS) in all the productions. This ensured high quality of the final radiotracer for all subsequent rodent experiments.

### 3.2 PET/CT imaging

**FIGURE 1:**
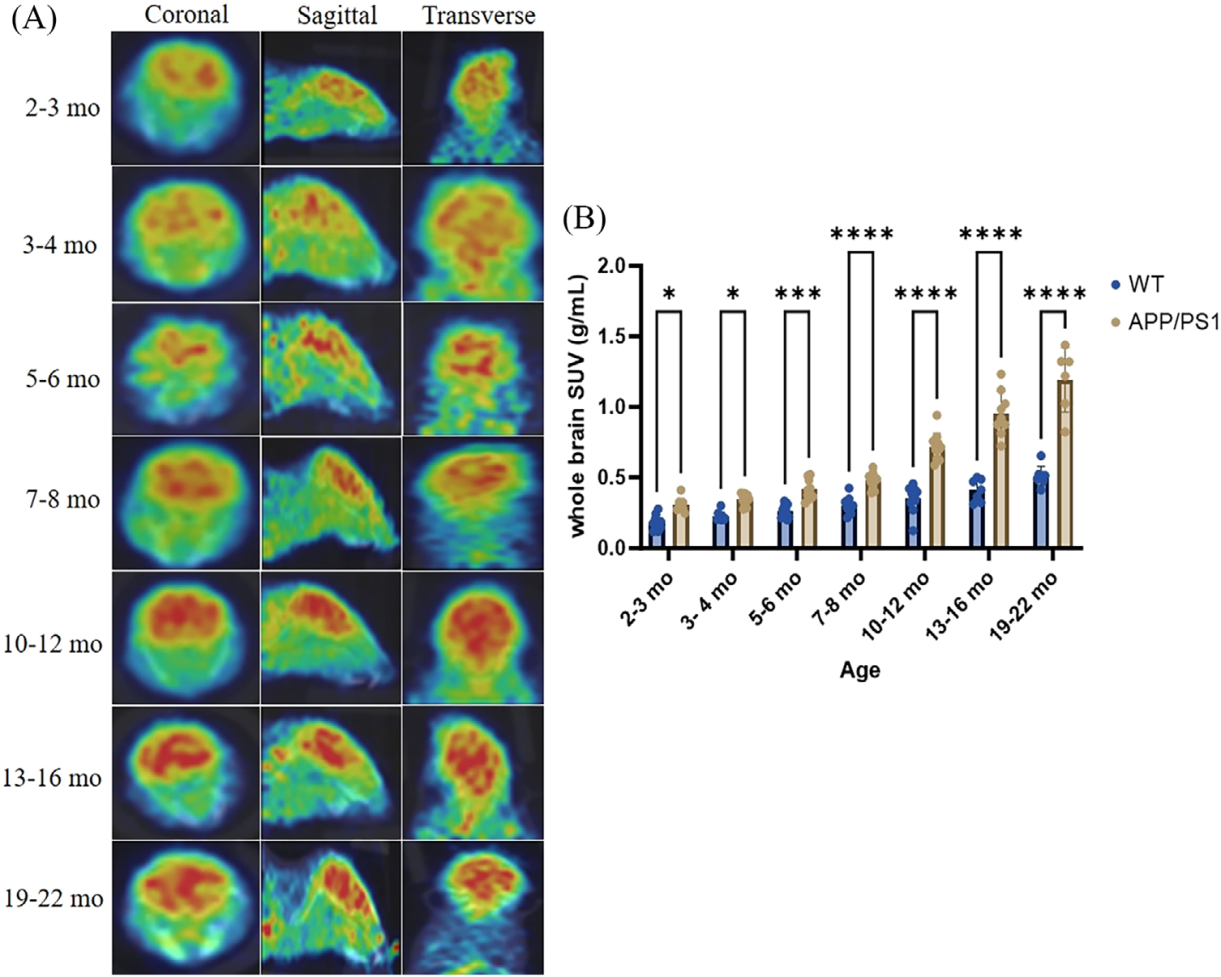
Longitudinal [^11^C]MPC‐6827 MT‐PET imaging was conducted on both APP/PS1 and WT mice. (A) Representative PET/CT images in coronal, sagittal, and horizontal views depict MT tracer [^11^C]MPC‐6827 uptake in APP/PS1 mice. Longitudinal analysis was performed at 2–3, 3–4, 5–6, 7–8, 10–12, 13–16, and 19–22 months of age. (B) Presents the whole‐brain SUV quantitative analysis. Data were representative of *n* = 7 from each group. Statistical analysis was performed using two‐way ANOVA followed by a Šídák's multiple comparisons test. Significant differences are denoted as ****p < 0.0001, ***p < 0.0002, **p < 0.0021, *p < 0.0332, and ns < 0.1234.

**FIGURE 2:**
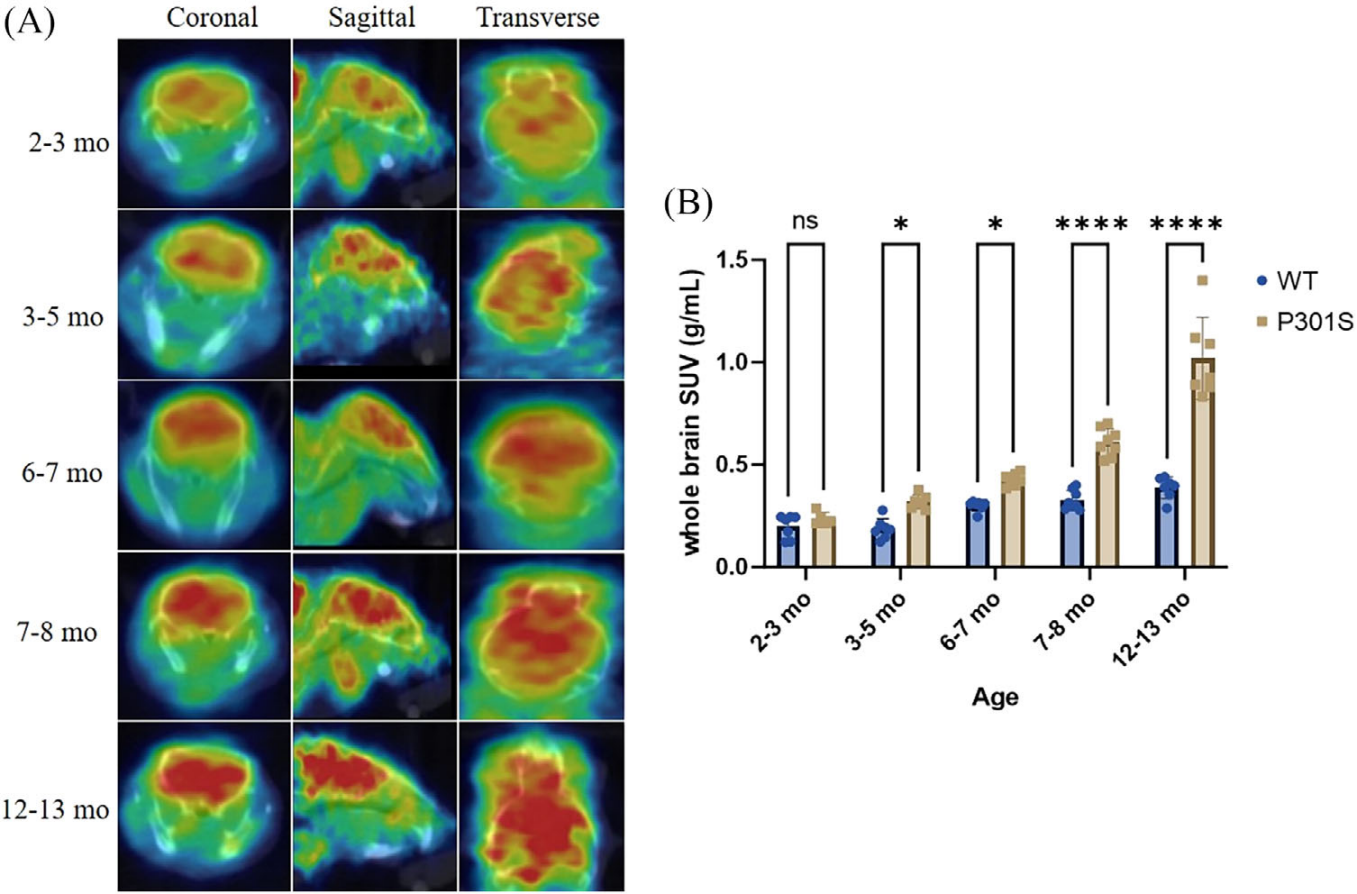
Longitudinal [^11^C]MPC‐6827 MT‐PET imaging was performed on both P301S and WT mice (*n* = 7 per group). (A) Depicts PET/CT images in coronal, sagittal, and horizontal views, illustrating tracer [^11^C]MPC‐6827 uptake in P301S mice. Longitudinal analysis was conducted at 2–3, 3–5, 6–7, 7–8, and 12–23 months of age. (B) Presents the whole‐brain SUV quantitative analysis. Statistical analysis was performed using two‐way ANOVA followed by a Šídák's multiple comparisons test in inter‐group analysis. Significant differences are denoted as ****p < 0.0001, ***p < 0.0002, **p < 0.0021, *p < 0.0332, and ns < 0.1234.

**FIGURE 3:**
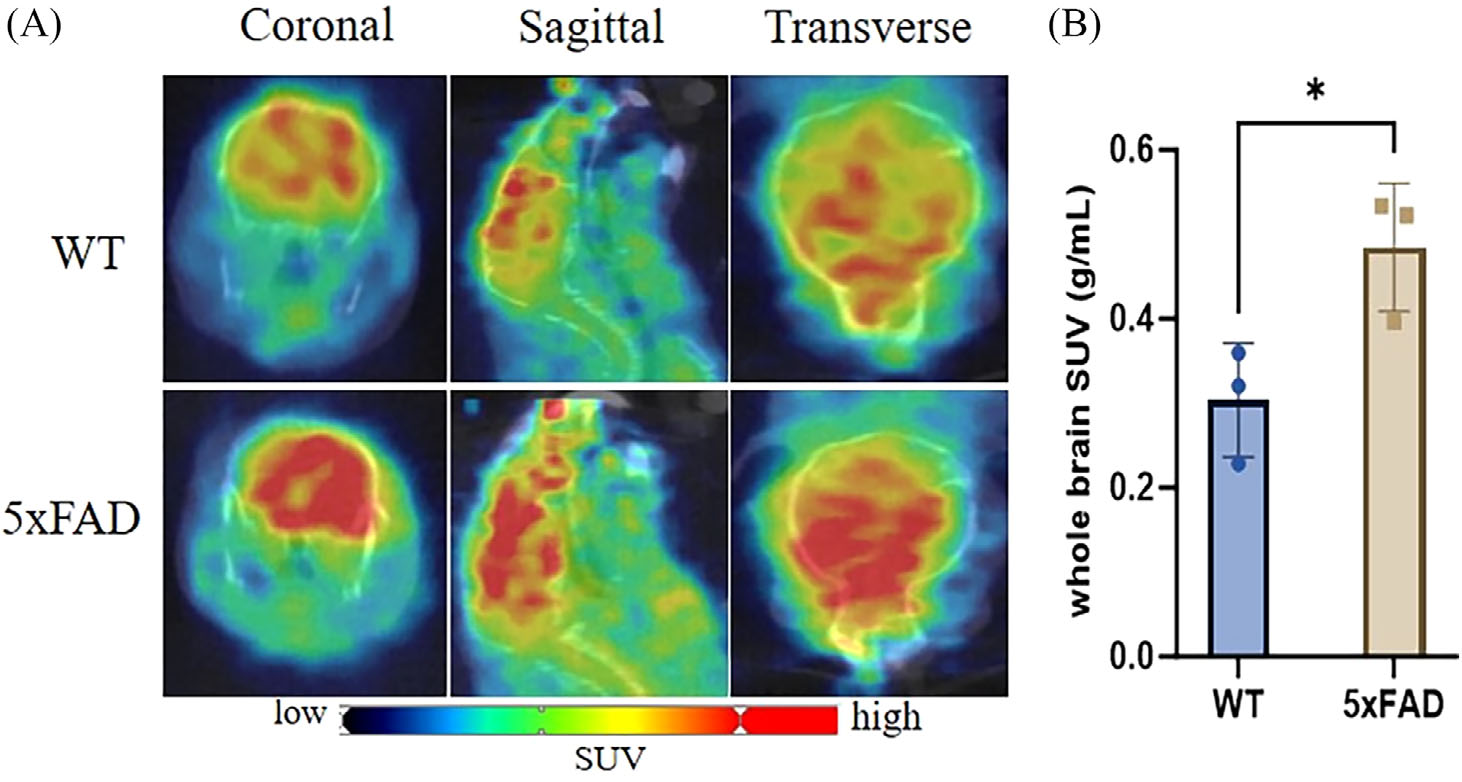
PET imaging was performed to evaluate [^11^C]MPC‐6827 uptake in 5xFAD mice. (A) Representative coronal, sagittal, and horizontal PET images depict 12‐month‐old 5xFAD and WT following [^11^C]MPC‐6827 tracer injection. (B) Whole‐brain SUV quantification of [^11^C]MPC‐6827 from 5xFAD and WT mice (*n* = 3/group) are presented. Statistical significance (*p ≤ 0.05) was observed in unpaired *t*‐test.

[^11^C]MPC‐6827 PET/CT (computerized tomography) imaging was performed in APP/PS1 and P301S mice, and in WT controls, across a range of age (2–22 months for APP/PS1; 2–13 months for P301S), as shown in Figures 1 and 2. Dynamic PET scans revealed a statistically significant increase in radiotracer uptake in whole‐brain regions of APP/PS1 mice compared to the WT mice, spanning from 2–3 months (*p = 0.0252), 3–4 months (*p = 0.0368), 5–6 months (***p = 0.0008),10–12 months (***p < 0.0001), 13–16 months (***p < 0.0001), and 19–22 months (***p < 0.0001), as illustrated in Figure 1A,B. P301S mice also showed a significant increase in brain radiotracer uptake at 3–5 months (*p = 0.0248), 6–7 months (*p = 0.0227), 7–8 months (****p = 0.0001), and 12–13 months (****p < 0.0001), as shown in Figure 2A,B. In transgenic mice, there was a 1.3‐fold higher uptake in APP/PS1 mice from 3 to 22 months and a 1.0‐fold higher uptake in P301S mice from 3 to 13 months. PET imaging with [^11^C]MPC‐6827 in 12‐month‐old 5xFAD mice also showed higher brain uptake compared to age‐matched WT littermates (*p = 0.0365; Figure 3A,B).

### 3.3 Biodistribution studies

**FIGURE 4:**
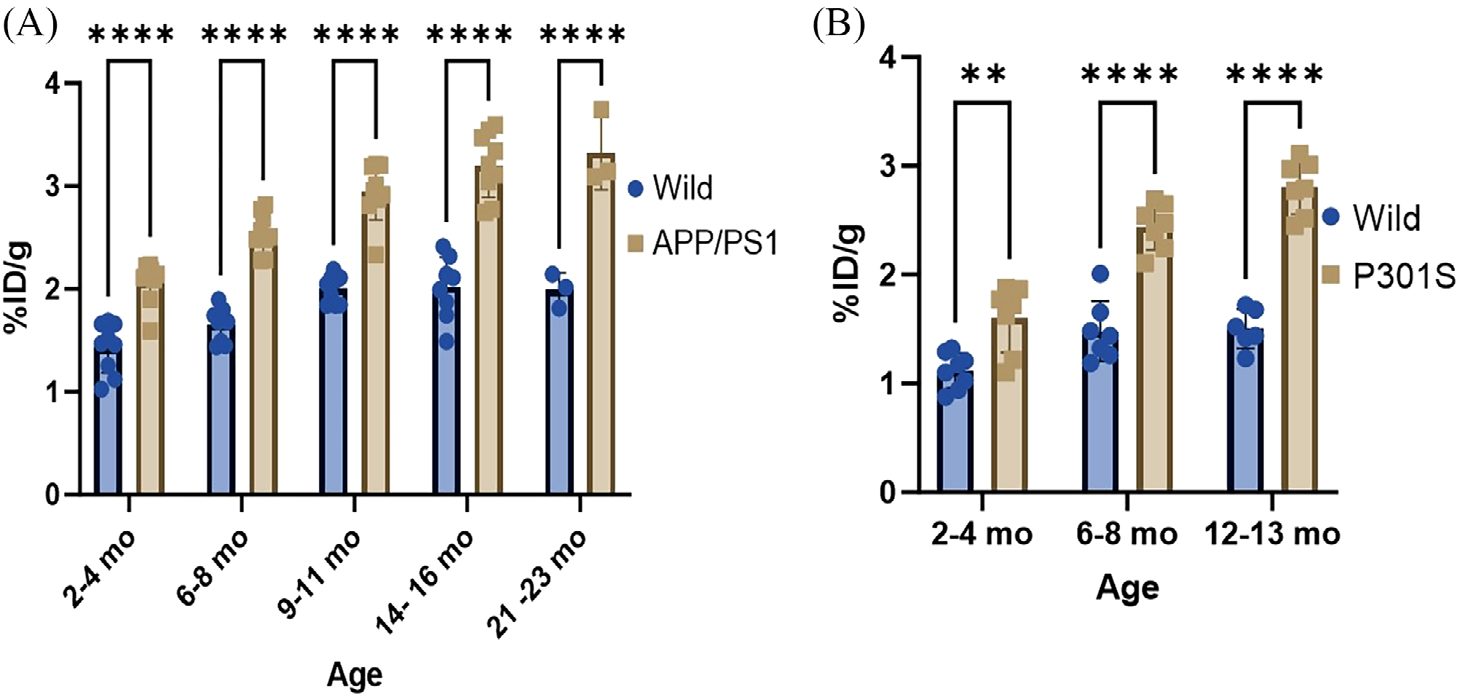
Longitudinal analysis of [^11^C]MPC‐6827 biodistribution in APP/PS1 and P301S mice. (A) Representative graph illustrating quantification (%ID/g) performed in the whole brain region of APP/PS1 and WT mice, plotted and analyzed at 2–4, 6–8, 9–11, 14–16, and 21–23 months of age. (B) Representative graph depicting quantification (%ID/g) in the whole brain region of P301S and WT mice, plotted and analyzed at 2–4, 6–8, and 12–13 months of age. The data, obtained from *n* = 8 animals per group, were analyzed using two‐way ANOVA followed by a Sidak's multiple comparisons test with significant differences are denoted as ****p < 0.0001, ***p < 0.0002, **p < 0.0021, *p < 0.0332, and ns < 0.1234.

Concurrent with PET/CT imaging, a separate cohort of APP/PS1 and P301S mice underwent ex vivo biodistribution studies at the corresponding PET imaging time points. These studies demonstrated significantly higher brain uptake in APP/PS1 and P301S mice compared to their age‐matched WT littermates (Figure 4). In APP/PS1 mice, radiotracer uptake was higher in the APP/PS1 brain compared to WT mice across all time‐points, that is, with a ****p‐value of <0.0001 at 2–4 months, 6–8 months, 10–12 months, 13–16 months, and 21–23 months (Figure 4A). P301S mouse brains also showed higher brain uptake at 2–4 months (**p = 0.0013), 6–8 months (****p = 0.0001), and 12–13 months compared to controls (****p < 0.0001, Figure 4B). Longitudinally, there was ≈1.2‐fold higher uptake in APP/PS1 mouse brains from 3 to 20 months and a 1.3‐fold higher uptake in P301S mouse brains from 2 to 13 months. Tables S1 and S2 show a full distribution profile of [^11^C]MPC‐6827 in both APP/PS1 and P301S mice including blood, heart, liver, lung, spleen, pancreas, kidney, and muscle.

### 3.4 Autoradiography

**FIGURE 5:**
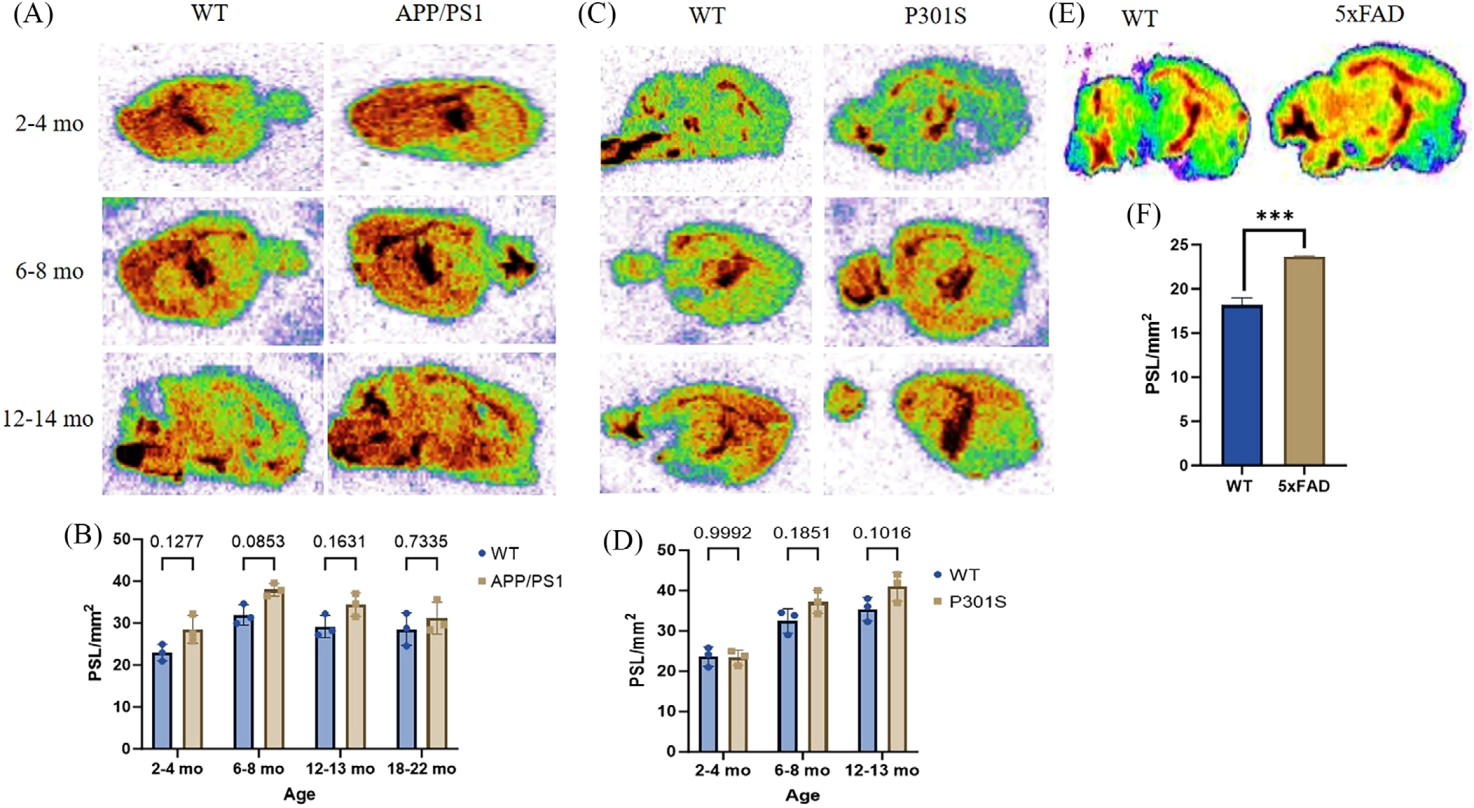
Longitudinal analysis of [^11^C]MPC‐6827 in vitro autoradiography in APP/PS1 and P301S mice. (A) Representative images of brain sections from WT (left) and APP/PS1 (right) mice at 3–4, 6–8, and 12–14 months of age. (B) Quantitative graph displaying photostimulated luminescence signals per square millimeter (PSL/mm^2^) of brain sections in APP/PS1 and WT mice. (C) Representative images of brain sections from WT (left) and P301S (right) mice at 2–4, 6–8, and 12–13 months of age. (D) Quantitative graph illustrating PSL/mm^2^ of brain sections in P301S and WT mice. The data, obtained from *n* = 3 animals per group, were analyzed using two‐way ANOVA followed by a Sidak's multiple comparisons test. (E) Representative autoradiography images depicting 12‐month‐old 5xFAD mice and WT counterparts. (F) Brain PSL/mm^2^ quantification of [^11^C]MPC‐6827 from 5xFAD and WT mice (*n* = 3/group). Statistical significance ***p ≤ 0.006) was observed in in unpaired *t*‐test.

The digital ARG studies were conducted on a subset of APP/PS1, P301S, and WT mice at various age points, as depicted in Figure 5. The radiotracer uptake was higher in APP/PS1 brain sections compared to WT, with *p*‐values of 0.1277, 0.0853, 0.1631, and 0.7335 at 2–4, 6–8, 12–13, and 18–22 months of age, respectively (Figure 5A,B). Similarly, radiotracer uptake in P301S brain sections showed higher uptake compared to WT brain samples, with corresponding *p*‐values of 0.9992, 0.1851, and 0.1016 at 2–4, 6–8, and 12–13 of age, respectively (Figure 5C,D). Furthermore, 5xFAD mouse brain sections also demonstrated high uptake compared to WT brain sections at 12 months of age, with a ***p‐value of 0.001 (Figure 5E,F).

### 3.5 Behavioral study

**FIGURE 6:**
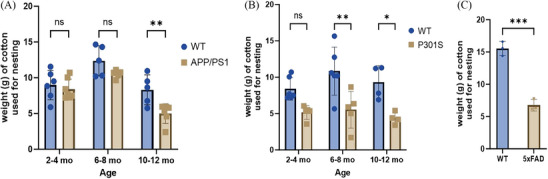
Nesting behavior study in the APP/PS1, P301S, and 5xFAD mouse cohorts. (A, B) Bar graph illustrating the results from nestlet nest construction experiments in APP/PS1, P301S, and their WT cohorts at different age points: 2–4, 6–8, and 10–12 months of age. (C) Bar graph depicting nesting behavior in WT and 5xFAD mice at 10–12 month of age. Data were representative of *n* = 6 in each cohort. Statistical analysis was performed using two‐way ANOVA followed by a Šídák's multiple comparisons test and unpaired *t*‐test.

Nest‐building behaviors in APP/PS1, P301S, 5xFAD, and WT mice served as their cognitive assessment and metric of self‐care.^27^, ^36^, ^37^ APP/PS1 mice demonstrated a slight decrease in nestlet use compared to WT controls by 10–12 months of age (**p‐value <0.0084), (Figure 6A). Similarly, P301S mice had alterations in behavior by 2–4 months, with reduced nestlet utilization relative to WT mice and *p*‐values of 0.0628, **0.0014, and *0.0109 at 2–4 months, 6–8 months, and 10–12 months, respectively (Figure 6B). Finally, 5xFAD TG mice also exhibited decreased nestlet use compared to WT mice at 12 months of age, with a ***p‐value of 0.001 (Figure 6C).

### 3.6 Quantitation of brain Aβ plaque and tau tangle depositions

Aβ and tau pathology was assessed in APP/PS1 and P301S mice using HJ3.4, an antibody against Aβ, and AT8, an antibody against hyperphosphorylated tau (p‐tau), respectively. Representative images of Aβ deposition demonstrate that Aβ pathology increases with age, beginning at 6–8 months in the hippocampus and cortex. By 18 months of age, widespread Aβ pathology is observed in these regions (see Figure S1A,B). Representative AT8‐stained p‐tau demonstrate that p‐tau is present in P301S mice as early as 2–3 months of age (*p* < 0.05) within the cortex and hippocampus and increases with age. AT8 staining begins as diffuse staining throughout the cortex and CA3 of the hippocampus at 2–4 months. More prominent cell body staining, indicative of neurofibrillary tangles, begins at 6–8 months and is widespread by 9–11 months (Figure S1C,D).

### 3.7 Free and bound tubulin dynamics

To delve into early MT dynamic changes, particularly assessing the percentage of free/destabilized tubulins, we conducted a western blot analysis in APP/PS1 and P301S mice relative to WT controls across different ages (see Figure S2). Results from APP/PS1 mice at 2, 6, and 12 months showed no significant differences in the free tubulin ratio compared to WT brains (see Figure S2A,B). However, APP/PS1 mouse brains exhibited less bound tubulin compared to age‐matched WT littermates, with *p*‐values of 0.003, 0.8216, and 0.6578 at 2, 6, and 12 months, respectively (see Figure S2C,D). Longitudinal analysis in P301S mice demonstrated more free/destabilized tubulin (see S2E) and less bound/stabilized tubulin (see Figure S2F) with *p*‐values of 0.3897, 0.994, and 0.0015 for free tubulin and *p*‐values of 0.7927, 0.7008, and 0.3518 for bound tubulin compared to age‐matched WT mouse brains (see Figure S2G,H), respectively.

## 4 DISCUSSION

The intricate involvement of destabilized MTs in neurodegenerative processes, particularly in AD and related dementia (ADRD) underscores their critical role in maintaining neuronal integrity and function. MTs serve as the primary tracks for axonal transport, contribute to neuronal structural stability, and modulate synaptic plasticity.^38^, ^39^ Destabilization or dysregulation of MT dynamics seems to be a common early neuronal change occurring across various neurodegenerative diseases, including AD. In AD, the pathological cascade involves the aggregation of Aβ and hyperphosphorylation of tau protein, leading to synaptic impairment, dendritic simplification, and neurodegeneration.^40^ Therefore, understanding the process of MT destabilization in the early stages of AD pathogenesis is essential due to its concurrence with Aβ and tau pathology, representing a pivotal role in disease progression.^41^ However, there is a significant gap in molecular diagnostic mechanisms capable of measuring MT dynamics and our understanding of how this relates to the primary pathologies in AD, amyloid and tau.

Literature suggests that the initiation of the formation of aggregatory prone, high‐order Aβ species and tau protein hyperphosphorylation typically occurs within a timeframe of 2 to 6 months of age in APP/PS1 and P310S mouse models.^42^ However, conventional molecular methods such as immunohistochemistry (IHC) detect Aβ aggregates and amyloid plaques closer to 6 months of age.^43^ Furthermore, currently used PET tracers like the [^11^]C‐PiB beta‐amyloid tracer show a significant increase in uptake at 12 months of age in APP/PS1 mice,^44^ whereas tau PET tracers like [^18^]F‐THK5351 demonstrate detectable signals at around 7 to 8 months of age in P301S mice.^45^ Although established rodent models including APP/PS1 and P301S allow for the detection of Aβ formation and tau tangle development, respectively, at different time points, information regarding the process of MT degeneration throughout their longitudinal life remains largely unknown.

To address this gap, our lab developed the first BBB‐penetrating MT PET radioligand, [^11^C]MPC‐6827, which demonstrated high affinity for destabilized MTs and excellent brain uptake in rodent and NHP models.^21^, ^22^ Leveraging this innovative approach, longitudinal studies in AD mouse models can provide invaluable insights into the temporal relationship between Aβ pathology, tau pathology, and MT destabilization. Our lab evaluated the mechanism of [^11^C]MPC‐6827 using MT stabilizing and destabilizing agents and concluded that the uptake was selective toward the MT destabilizing environment.^24^ We then successfully explored the high translational PET imaging utility of [^11^C]MPC‐6827 in an aging NHP model with AD‐related pathology.^25^ Our preliminary data on increase in radiotracer uptake with AD burden complement other research group findings including those of Lindberg et al., who showed higher radiotracer uptake of [^11^C]MPC‐6827 in human AD brain tissues compared to age‐matched healthy controls.^46^ We also showed [^11^C]MPC‐6827's excellent CNS‐based PET radiotracer properties^23^ including high serum and metabolic stability in rodent brain and plasma samples and a favorable dissociation constant (K_d_) of 15.59 nM with specific binding constant (B_max_) of 11.86 fmol/mg.

In this project, to explore the early imaging utility of [^11^C]MPC‐6827 in AD, our comprehensive longitudinal analysis employed [^11^C]MPC‐6827 PET imaging in APP/PS1 and P301S mouse models compared to WT controls. This study encompassed a range of ages, from 2 to 22 months for APP/PS1 mice and 2 to 13 months for P301S mice and was supplemented by intra‐group and inter‐group comparisons. Our inter‐group study revealed an increase in [^11^C]MPC‐6827 brain uptake emerging from 2 months onward, with significant changes in tracer uptake becoming evident from 6 months of age in both APP/PS1 and P301S mice compared to WT mice. These findings suggest an early onset of MT dysregulation, with [^11^C]MPC‐6827 effectively tracking these changes in real time and in vivo.

In‐depth comprehensive longitudinal analysis, focusing on APP/PS1 and P301S mice at different age points, allowed us to perform intra‐group analyses, providing critical insights into the dynamic changes associated with AD progression. Our findings revealed statistically significant differences in the APP/PS1 mouse models, both prior to Aβ deposition during amyloid plaque formation with *p*‐values of ***0.003 and ****0.0001, respectively. Our MT tracer shows changes in the APP/PS1 brain earlier than changes can be detected with amyloid PET. Similarly, in the P301S mice, significant differences were observed at both the early ptau formation stage (2 to 6 months of age) and the histological tau deposition stage (7 to 22 months of age), with *p*‐values of ***0.007 and ****0.0001, respectively. Consistently, biodistribution in brain samples from APP/PS1 and P301S mice corroborated with the PET results, validating our PET observations. Our preliminary PET imaging analysis in a smaller sample size (*n* = 2/group) showed lower brain radiotracer uptake in the very old 22 month old APP/PS1 and 18 month old P301S TG over WT mice.^47^ However, in this report our rigorous and comprehensive intra‐group longitudinal analysis (*n* = 8/group) revealed a statistically significant progressive increase in MT dysregulation with increasing in AD loads, highlighting the utility of the [^11^C]MPC‐6827 tracer in detecting MT‐based alterations associated with AD progression.

**FIGURE 7:**
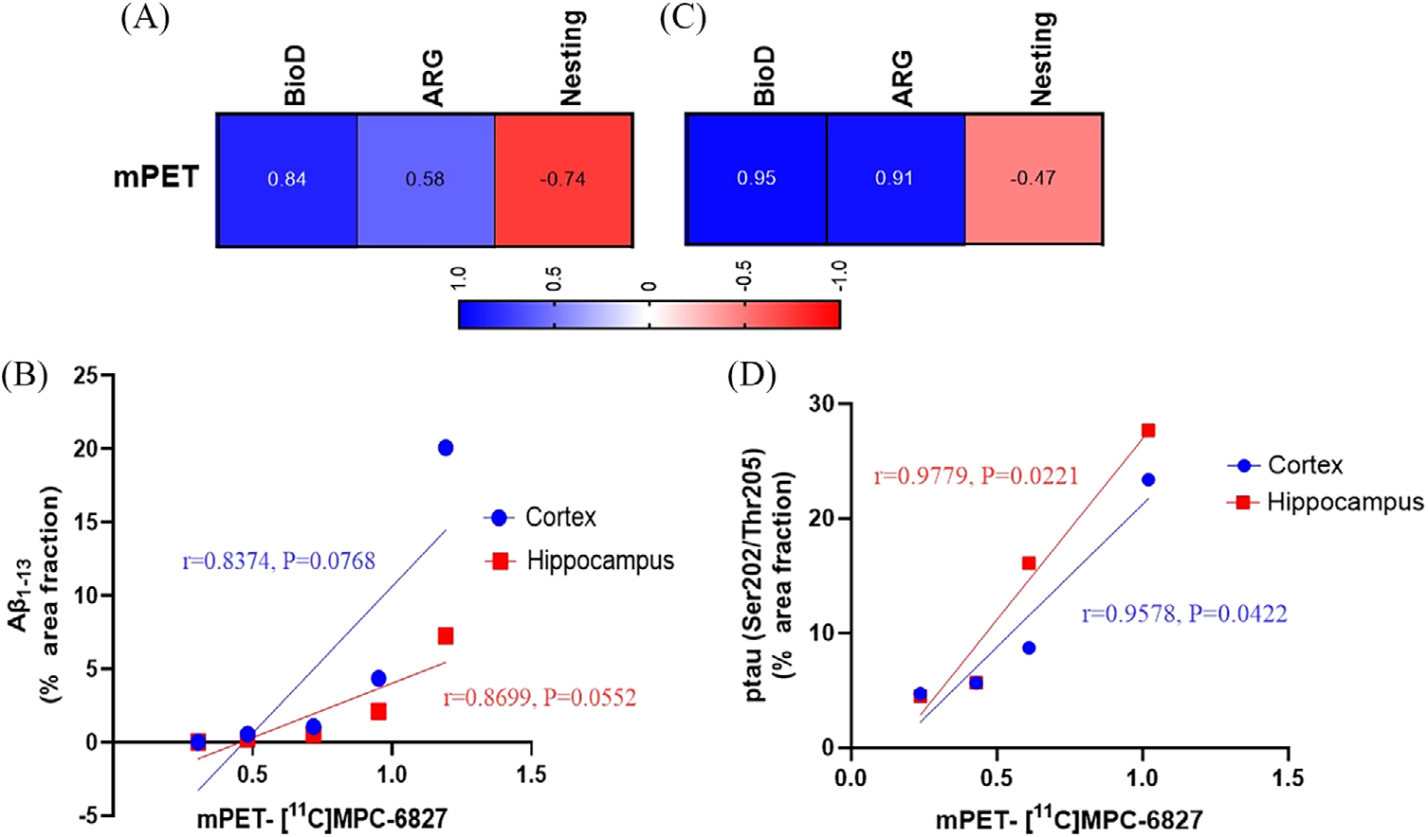
Correlation analysis of [^11^C]MPC‐6827 among microPET imaging, biodistribution, nesting behavior, and autoradiography studies. (A, C) Present the Pearson correlation coefficients (*R*) heatmap for (A) APP/PS1 and (C) P301S cohorts. (B, D) Scattered plots illustrating the relationship between (B) PET signal (SUV) and Aβ_1‐13_ (% cortex area and hippocampus) and (C) PET signal (SUV) and phospho‐tau (AT8) (% cortex area and hippocampus) at 2–4, 6–8, and 10–12 months of age. The data points were fitted by linear regression, indicating a high Pearson *r*‐value and statistically positive correlation. Data analyzed with Newman–Keuls post hoc test.

In our study, we performed a correlation analysis integrating data from PET/CT imaging, biodistribution, autoradiography, behavioral (nest building), and immunohistochemistry (Aβ and tau pathology). In APP/PS1 mice, robust positive correlations were observed between PET data and biodistribution (Pearson *r* = 0.84), autoradiography results (Pearson *r* = 0.58), and Aβ_1‐13_ ‐cortex (Pearson *r* = 0.8374) and Aβ_1‐13_ ‐Hippocampus (Pearson *r* = 0.8699) (Figure 5A,B). Similarly, in P301S mice, PET findings positively correlated with biodistribution data (Pearson *r* = 0.95) and autoradiography data (Pearson *r* = 0.91) and p‐tau (AT8) ‐cortex (Pearson *r* = 0.9578) and p‐tau (AT8) ‐Hippocampus (Pearson *r* = 0.9779) (Figure 5C,D). These strong significant positive correlations underscore the interplay of MT destabilization, amyloid, and tau pathology in AD pathogenesis, highlighting the congruence between molecular pathology and imaging outcomes. Notably, a negative correlation was observed between PET and behavioral study data in both APP/PS1 (Pearson *r* = −0.74) and P301S (Pearson *r* = −0.47) cohorts. Furthermore, analysis of Figure 6 unveiled disparities in nestlet use between APP/PS1 and P301S mice compared to their respective WT counterparts at specific ages. APP/PS1 mice exhibited significantly lower nestlet use at ages 10–12 months, whereas P301S mice displayed significantly lower nestlet use at ages 6–8 months. This is consistent with human AD, where behavioral changes are more associated with tau pathology than amyloid. These findings suggest the potential utility of [^11^C]MPC‐6827 MT PET imaging in detecting AD‐related changes at early stages preceding observable behavioral alterations (Figure 7).

Furthermore, the inclusion of the 5xFAD mouse model, renowned for its extensive Aβ plaque deposition and aggregation, synaptic dysfunction, and neuroinflammation,^48^ adds a crucial dimension to our investigation. Notably, this mouse model lacks human tau transgene expression; yet it exhibits tau hyperphosphorylation.^49^ The radiotracer demonstrated a 0.76‐fold higher uptake observed in 12‐month‐old 5xFAD mice compared to age‐matched WT mice, alongside its correlations with Aβ_42_ and resultant behavioral changes, underscoring the efficacy of [^11^C]MPC‐6827 in delineating MT dysregulation induced by Aβ toxicity. Moreover, we evaluated MT disintegration in AD by using traditional western blot techniques to quantify free and bound tubulin in APP/PS1 and P301S mice at different ages. However, these standard analyses detected only minimal differences in the bound and free tubulin ratios longitudinally (Figure S2). This suggests that [^11^C]MPC‐6827 PET imaging may emerge as a highly sensitive method for studying MT dynamics.

Overall, the microPET imaging, biodistribution, autoradiography, and behavioral nesting results are very well corroborated, and our findings suggest the potential of [^11^C]MPC‐6827 PET imaging for detecting early MT alterations in established Aβ‐ and tau‐overexpressing rodent models of AD. These results enhance our understanding of AD progression and lay the groundwork for improved diagnostic and therapeutic strategies.

Alterations in MT dynamics are prevalent in numerous neurodegenerative disorders, including Parkinson's disease, hereditary spastic paraplegia, amyotrophic lateral sclerosis, and Huntington's disease.^50^ The application of [^11^C]MPC‐6827 PET imaging extends beyond AD, offering insights into the pathophysiological changes associated with these conditions. Given that many therapeutic interventions target MT stability, [^11^C]MPC‐6827 has the potential to monitor the efficacy of treatments. For example, it could be utilized to evaluate therapies involving epothilone D, which modulates MT stability.^51^ As MT‐targeting agents undergo increasing evaluation in neurodegeneration models and progress through clinical trials, the capability of [^11^C]MPC‐6827 to visualize and quantify changes in MT dynamics may facilitate the development and optimization of novel pharmacological interventions, thereby potentially enhancing clinical outcomes for patients with various neurodegenerative diseases.

Future research should address the limitations of our study, including conducting sex‐based analysis and directly correlating findings with existing PET tracers targeting Aβ and tau pathology and CSF biomarkers (CSF Aβ_1‐42_, CSF p‐tau, CSF t‐tau [total tau]) and neurodegeneration blood markers (neurofilament light chain (NFL), neurogranin). Human clinical trials could further assess the efficacy of [^11^C]MPC‐6827 in detecting MT changes in patients with AD.

## AUTHOR CONTRIBUTIONS

Kiran K. Solingapuram Sai conceived, designed, and executed the project. Naresh Damuka, Riley E. Irmen, Krishna K. Gollapelli, Ivan Krizan, and Bhuvanachandra Bhoopal conducted experiments including radiochemistry, rodent imaging, biodistribution, autoradiography, behavioral studies, immunohistochemistry, and tubulin assays. Mack Miller and Ojasvi Deep analyzed PET/CT images. Data analysis and interpretation were carried out by Kiran K. Solingapuram Sai, Naresh Damuka, Shannon L. Macauley, Avinash Bansode, Miranda E. Orr, Akiva Mintz, Samuel N. Lockhart, Christopher T. Whitlow, and Suzanne Craft. Kiran K. Solingapuram Sai and Naresh Damuka compiled the manuscript, which was approved by all authors.

## CONFLICT OF INTEREST STATEMENT

The authors declare no conflicts of interest. Author disclosures are available in the supporting information.

## Supporting information

Supporting Information

Supporting Information

Supporting Information

Supporting Information

Supporting Information
