# Supplementary material for: Exploring microtubule dynamics in Alzheimer's disease: Longitudinal assessment using [11C]MPC‐6827 PET imaging in rodent models of Alzheimer's‐related pathology
Source: Alzheimers Dement. 2024 Jul 5;20(9):6082–93. doi: 10.1002/alz.14083 (PMC11497705; doi:10.1002/alz.14083)
Supplement: Supplementary file 1 — Supporting Information [file ALZ-20-6082-s004.docx]

**Supporting data**

**Exploring microtubule dynamics in Alzheimer's disease: Longitudinal assessment using [^11^C]MPC-6827 PET imaging in rodent models of Alzheimer’s-related pathology**

Naresh Damuka^1^, Riley E. Irmen^2^, Ivan Krizan^1^, Mack Miller^1^, Krishna Gollapelli^1^, Bhuvanachandra Bhoopal^1^, Ojaswi Deep^1^, Avinash Bansode,^1^ Samuel N. Lockhart^3^, Miranda Orr^3^, Pooja Jadiya^3^, Nagaraju Bashetti^4^, JV Shanmukha Kumar^4^, Akiva Mintz^5^, Christopher T Whitlow^1^, Shannon L Macauley^2^, Suzanne Craft^3^, Kiran K Solingapuram Sai^1*^

^1^Department of Radiology, Wake Forest School of Medicine, Winston-Salem, NC, 27157, USA.
^2^University of Kentucky, Department of Physiology, Lexington, KY, 40536, USA.

^3^Department of Gerontology and Geriatric Medicine, Wake Forest School of Medicine, Winston-Salem, NC, 27157, USA.

^4^Department of Chemistry, Koneru Lakshmaiah Education Foundation, Vijayawada, Andhra Pradesh, 522302, India.

^5^Department of Radiology, Columbia University Medical Center, New York, 10032, USA.

Correspondence

Kiran K Solingapuram Sai, Wake Forest School of Medicine, Department of Radiology, Winston-Salem, NC, 27157, USA.

Email: [ksolinga@wakehealth.edu](mailto:ksolinga@wakehealth.edu)

**Supplementary Table 1:** Biodistribution profile of [^11^C]MPC-6827 in APP/PS1 mice.

| **APP/PS1** | **2-4 (mo)** | | | | **6-8 (mo)** | | | | **9-11 (mo)** | | | | **14-16 (mo)** | | | |
| --- | --- | --- | --- | --- | --- | --- | --- | --- | --- | --- | --- | --- | --- | --- | --- | --- |
|  | **WT** | | **APP/PS1** | | **WT** | | **APP/PS1** | | **WT** | | **APP/PS1** | | **WT** | | **APP/PS1** | |
|  | **%ID/g** | **SD** | **%ID/g** | **SD** | **%ID/g** | **SD** | **%ID/g** | **SD** | **%ID/g** | **SD** | **%ID/g** | **SD** | **%ID/g** | **SD** | **%ID/g** | **SD** |
| blood | **1.29** | 0.81 | **1.33** | 0.45 | **1.33** | 0.46 | **1.24** | 0.15 | **1.79** | 0.57 | 1.46 | 0.21 | **1.24** | 0.21 | **1.56** | 0.37 |
| heart | **1.21** | 0.72 | **1.56** | 0.89 | **1.70** | 0.23 | **1.65** | 0.81 | **1.45** | 0.16 | 1.78 | 0.81 | **2.11** | 0.51 | **1.70** | 0.89 |
| liver | **6.21** | 1.48 | **6.98** | 1.56 | **7.22** | 2.01 | **6.11** | 2.11 | **6.87** | 1.69 | 7.98 | 1.87 | **5.65** | 1.12 | **6.90** | 1.36 |
| lung | **2.46** | 1.25 | **1.56** | 0.65 | **1.43** | 0.87 | **2.11** | 0.77 | **2.01** | 0.57 | 2.22 | 1.12 | **1.56** | 0.13 | **1.52** | 0.55 |
| spleen | **1.07** | 0.35 | **0.90** | 0.21 | **0.79** | 0.12 | **0.97** | 0.11 | **1.03** | 0.21 | 0.87 | 0.21 | **0.87** | 0.13 | **1.12** | 0.74 |
| pancreas | **2.44** | 0.88 | **1.88** | 0.13 | **1.89** | 0.11 | **0.78** | 0.21 | **1.24** | 0.41 | 1.53 | 0.14 | **0.88** | 0.12 | **1.24** | 0.24 |
| kidney | **8.70** | 1.22 | **9.32** | 2.31 | **10.22** | 2.11 | **9.43** | 3.22 | **9.21** | 3.13 | 10.11 | 2.45 | **9.53** | 1.24 | **10.29** | 3.21 |
| muscle | **0.89** | 0.27 | **0.74** | 0.11 | **0.55** | 0.09 | **0.65** | 0.19 | **0.59** | 0.09 | 0.45 | 0.08 | **0.26** | 0.06 | **0.42** | 0.10 |

**Supplementary Table 2:** Biodistribution profile of [^11^C]MPC-6827 in P301S mice.

| **P301S** | **2-4 (mo)** | | | | **6-8 (mo)** | | | | | **12-13 (mo)** | | | | |
| --- | --- | --- | --- | --- | --- | --- | --- | --- | --- | --- | --- | --- | --- | --- |
|  | **WT** | | **P301S** | | **WT** | | **P301S** | | | **WT** | | **P301S** | | |
|  | **%ID/g** | **SD** | **%ID/g** | **SD** | **%ID/g** | **SD** | **%ID/g** | **SD** | **%ID/g** | | **SD** | **%ID/g** | **SD** |  |
| blood | **0.93** | 0.25 | **1.26** | 0.31 | **1.12** | 0.46 | **1.54** | 0.12 | **1.32** | | 0.54 | **1.10** | 0.27 |  |
| heart | **1.74** | 0.21 | **1.69** | 0.46 | **2.12** | 0.87 | **1.98** | 0.53 | **1.12** | | 0.41 | **1.79** | 0.37 |  |
| liver | **5.98** | 1.48 | **6.24** | 1.37 | **6.55** | 1.23 | **5.44** | 1.10 | **7.12** | | 1.37 | **7.46** | 2.23 |  |
| lung | **3.04** | 0.31 | **4.13** | 1.22 | **2.15** | 0.88 | **3.65** | 1.94 | **2.90** | | 1.37 | **3.43** | 1.02 |  |
| spleen | **1.12** | 1.02 | **0.90** | 0.45 | **0.48** | 0.26 | **0.75** | 0.46 | **0.75** | | 0.54 | **0.81** | 0.26 |  |
| pancreas | **1.29** | 0.79 | **1.13** | 0.46 | **1.12** | 0.54 | **1.52** | 0.26 | **0.52** | | 0.12 | **1.12** | 0.84 |  |
| kidney | **10.50** | 4.24 | **9.79** | 2.36 | **11.25** | 2.45 | **10.41** | 5.12 | **11.22** | | 4.51 | **11.58** | 3.70 |  |
| muscle | **0.32** | 0.11 | **0.54** | 0.10 | **0.12** | 0.06 | **0.42** | 0.16 | **0.32** | | 0.06 | **0.19** | 0.11 |  |

**Note:** The brain uptake data is represented in Figure 4 of the main manuscript.

**Supplementary Figure 1:** Immunohistological analysis of amyloid and tau deposition


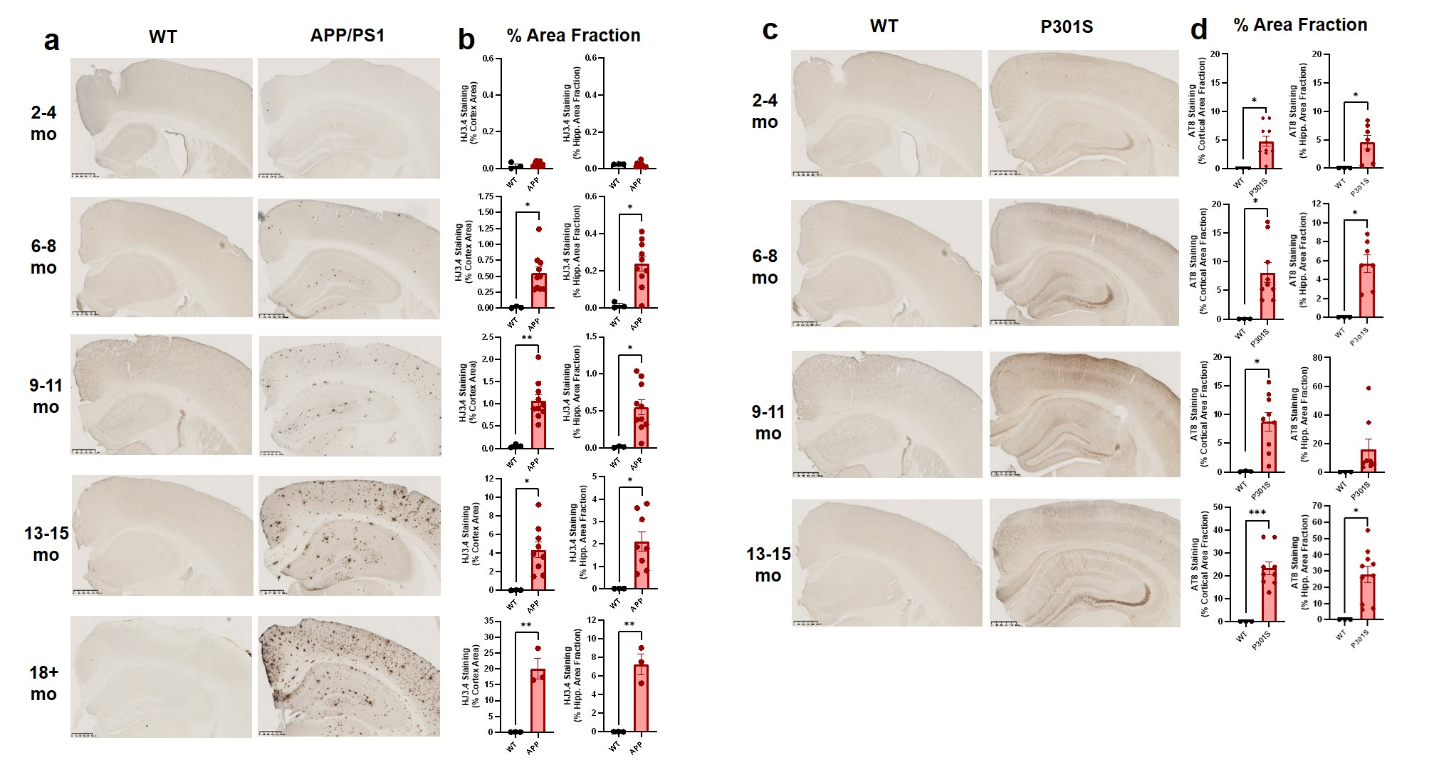


**Supplementary Figure 1**: Quantification of amyloid deposition and phosphorylated tau in APP/PS1 and P301S PS19 mice. (a) Representative images of Aβ deposition stained with HJ3.4 mouse monoclonal antibody against Aβ in APP/PS1 and WT mice at 2-4 months, 6-8 months, 9-11 months, 13-15 months, and 18+ months old. (b) Aβ deposition in the cortex and hippocampus was quantified and represented as area fraction. Aβ deposition is increased in the cortex and hippocampus as early as 6-8 months old (p<0.05) and increases as the mice age. (c) Representative images of phosphorylated tau stained with AT8 monoclonal antibody in P301S and WT mice at 2-4 months, 6-8 months, 9-11 months, and 13-15 months old. (d) Phosphorylated tau is present in P301S mice as early as 2-3 months old (p<0.05) within the cortex and hippocampus and increases with age. AT8 staining begins as diffuse staining throughout the cortex and CA3 of the hippocampus at 2-4 months. More prominent cell body staining, indicative of neurofibrillary tangles, begins at 6-8 months and is widespread by 9-11 months. p<0.05*, p<0.01**, p<0.001***. Data analyzed with student’s t-test and represented as means ± SEM. N=3-6 per group.

**Supplementary Figure 2:** Western blot analysis


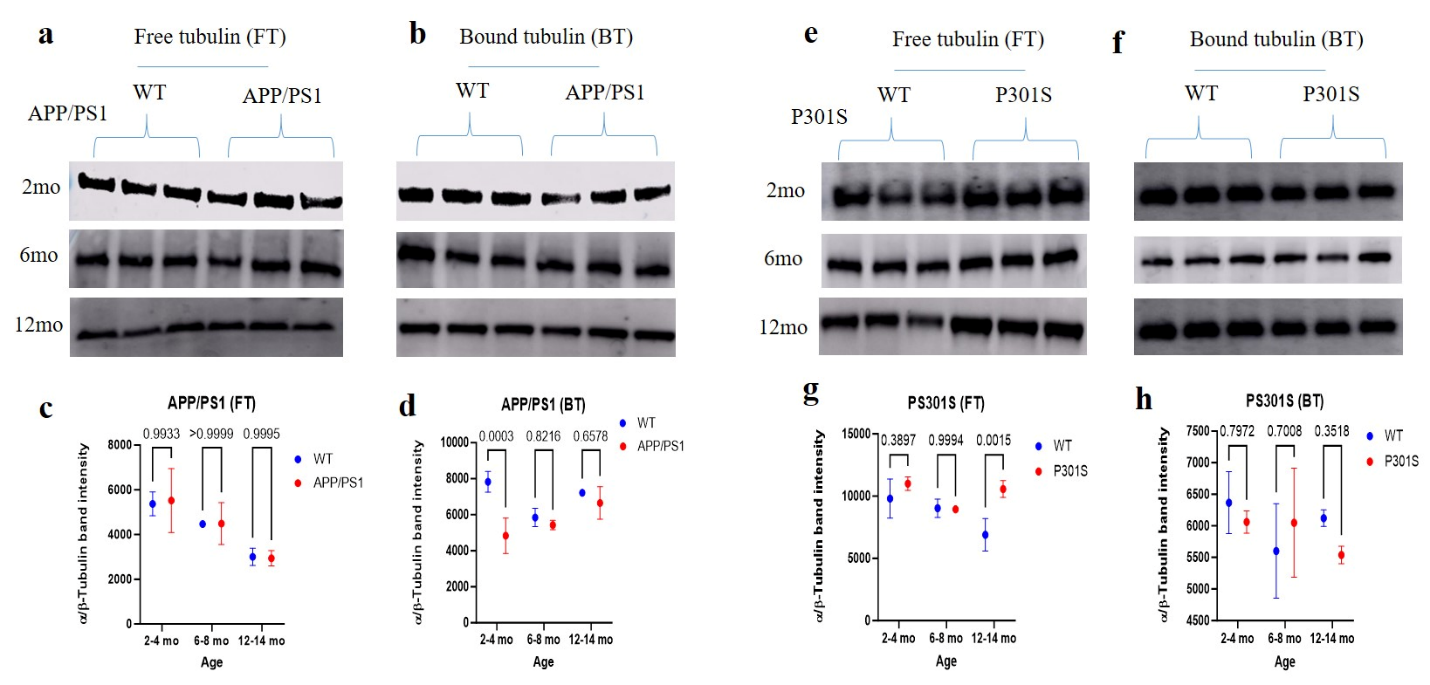


**Supplementary Figure 2**: Western blot analysis was conducted to assess the levels of free and bound tubulin in both WT and TG mice of the APP/PS1 and P301S strains across various age points. Representative blots illustrating free tubulin in APP/PS1 (a) and P301S (e) mice, as well as bound tubulin in APP/PS1 (b) and P301S (f) mice, are presented. Intensity quantification of free and bound tubulin blots for both APP/PS1 (c-d) and P301S (g-h) mice was conducted. The data, obtained from three animals per group, were analyzed using one-way ANOVA followed by a Newman-Keuls post hoc test.
