# Supplementary figures and images for: Exploring microtubule dynamics in Alzheimer's disease: Longitudinal assessment using [11C]MPC‐6827 PET imaging in rodent models of Alzheimer's‐related pathology
Source: Alzheimers Dement. 2024 Jul 5;20(9):6082–93. doi: 10.1002/alz.14083 (PMC11497705; doi:10.1002/alz.14083)

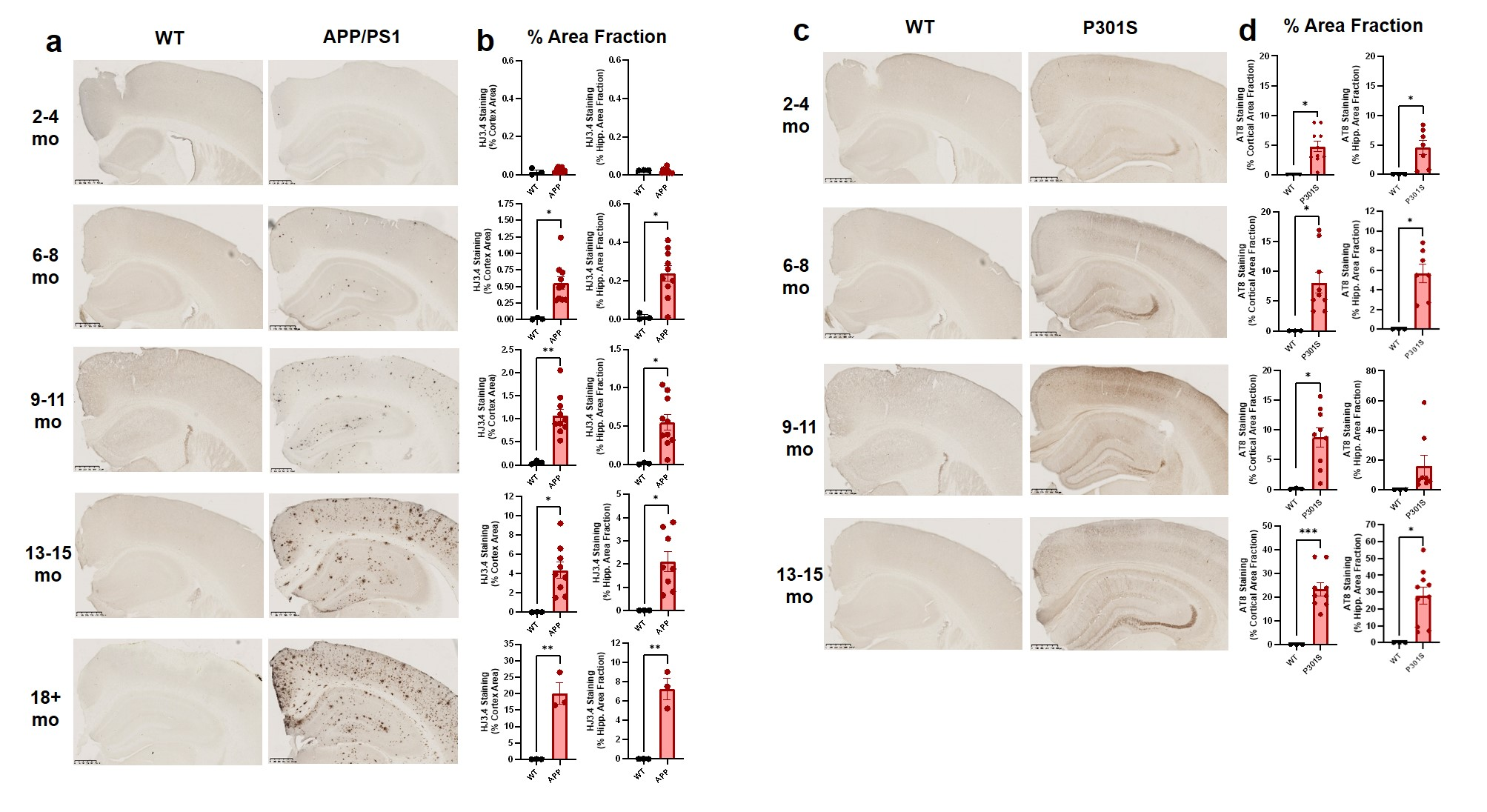

Supplement: Supplementary file 3 — Supporting Information [file ALZ-20-6082-s002.tif]

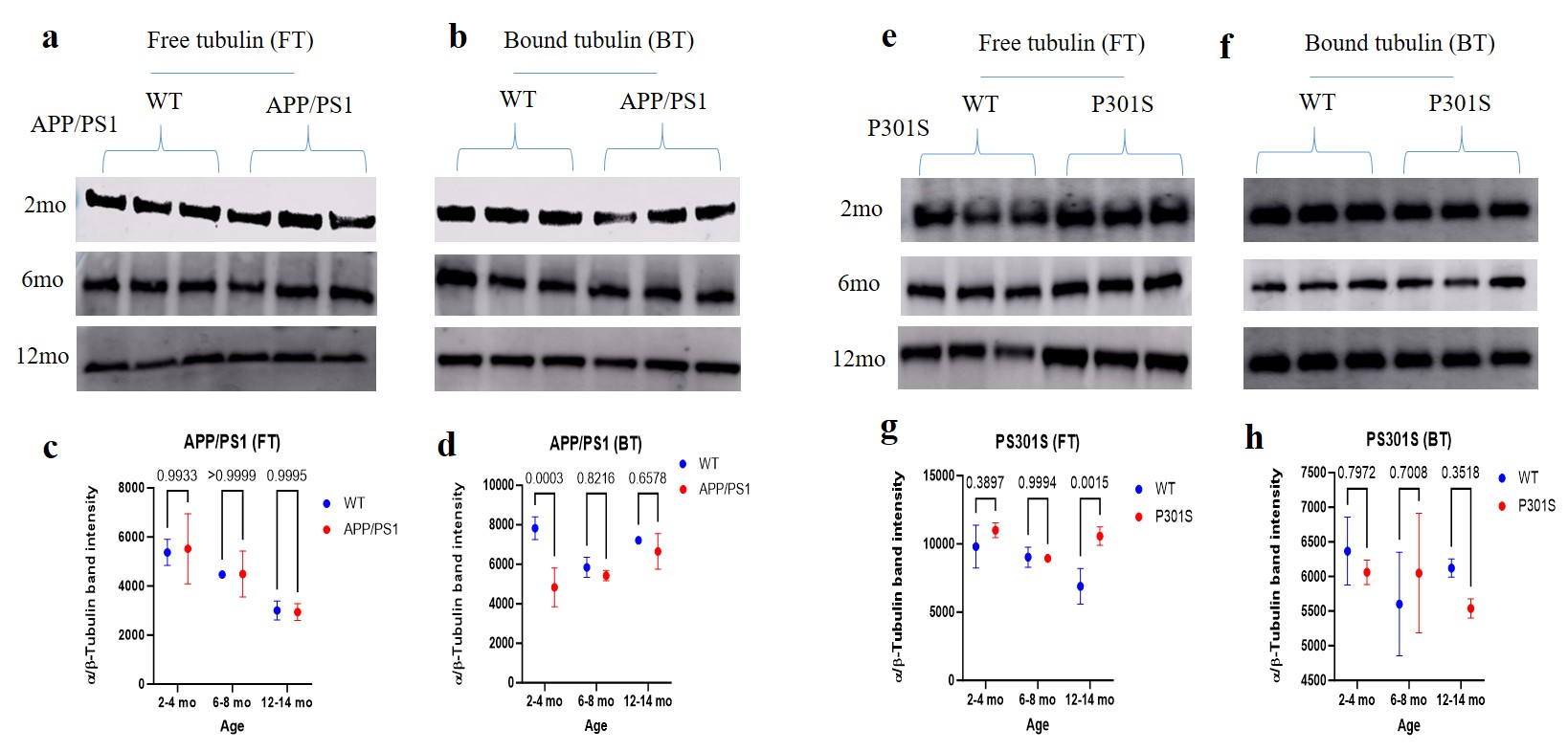

Supplement: Supplementary file 4 — Supporting Information [file ALZ-20-6082-s001.tif]
